# Linking properties of an orb‐weaving spider's capture thread glycoprotein adhesive and flagelliform fiber components to prey retention time

**DOI:** 10.1002/ece3.5525

**Published:** 2019-08-15

**Authors:** Brent D. Opell, Cassandra M. Burba, Pritesh D. Deva, Matthew H. Y. Kin, Malik X. Rivas, Hannah Mae Elmore, Mary L. Hendricks

**Affiliations:** ^1^ Department of Biological Sciences Virginia Tech Blacksburg VA USA

**Keywords:** Argiope aurantia, Argiope trifasciata, biological adhesive, environmental responsiveness, hygroscopic material, prey retention

## Abstract

1. An orb web's adhesive capture spiral is responsible for prey retention. This thread is formed of regularly spaced glue droplets supported by two flagelliform axial lines. Each glue droplet features a glycoprotein adhesive core covered by a hygroscopic aqueous layer, which also covers axial lines between the droplets, making the entire thread responsive to environmental humidity.
2. We characterized the effect of relative humidity (RH) on ability of *Argiope aurantia* and *Argiope trifasciata* thread arrays to retain houseflies and characterize the effect of humidity on their droplet properties. Using these data and those of *Araneus marmoreus* from a previous study, we then develop a regression model that correlated glycoprotein and flagelliform fiber properties with prey retention time. The model selection process included newly determined, humidity‐specific Young's modulus and toughness values for the three species' glycoproteins.
3. Argiope *aurantia* droplets are more hygroscopic than *A. trifasciata* droplets, causing the glycoprotein within *A. aurantia* droplets to become oversaturated at RH greater than 55% RH and their extension to decrease, whereas *A. trifasciata* droplet performance increases to 72% RH. This difference is reflected in species' prey retention times, with that of *A. aurantia* peaking at 55% RH and that of *A. trifasciata* at 72% RH.
4. Fly retention time was explained by a regression model of five variables: glue droplet distribution, flagelliform fiber work of extension, glycoprotein volume, glycoprotein thickness, and glycoprotein Young's modulus.
5. The material properties of both glycoprotein and flagelliform fibers appear to be phylogenetically constrained, whereas natural selection can more freely act on the amount of each material invested in a thread and on components of the thread's aqueous layer. Thus, it becomes easier to understand how natural selection can tune the performance of viscous capture threads by directing small changes in these components.

## 1 INTRODUCTION

### 1.1 Orb web prey capture threads and the challenge of understanding their performance

**Figure 1:**
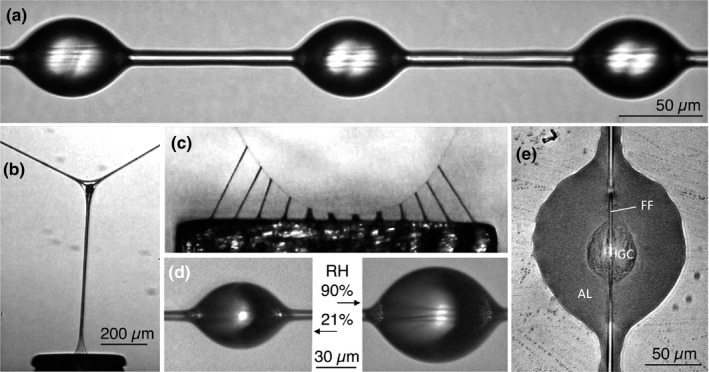
Viscous capture threads and droplets. (a) *Argiope trifasciata* thread. (b) An extending *A. trifasciata* droplet being pulled from a probe. The glycoprotein core and supporting axial fibers are visible within the aqueous layer at the thread‐support line junction. (c) A capture thread strand being pulled from a 2‐mm‐wide surface. (d) The same *A. aurantia* droplet photographed at low and high relative humidities (RH). (e) An *A. aurantia* droplet flattened to show its glycoprotein core (GC), surrounding aqueous layer (AL), and contiguous flagelliform fiber support lines (FF)

Animals use adhesives for many purposes. For example, insects glue their eggs to both wet and dry surfaces (Gaino & Mazzini, 2009; Li, Huson, & Graham, 2008), mussels and barnacles attach to marine substrates (Dickinson et al., 2009; Kamino, 2010; Naldrett, 1993; So et al., 2016; Waite, 2017), caddis fly larvae and some polychaete annelids build tubes from sand and gravel (Mackay & Wiggins, 1979; Shcherbakova, Tzetlin, Mardashova, & Sokolova, 2017; Wang, Svendsen, & Stewart, 2010), and sea cucumbers expel adhesive Cuvierian tubules for defense (Baranowska, Schloßmacher, McKenzie, Müller, & Schröder, 2011; Flammang & Becker, 2010; Flammang, Ribesse, & Jangoux, 2002). After being secreted as low viscosity solutions, these adhesives stiffen to resist crack propagation that leads to failure. In contrast, the glue droplets of an araneoid orb‐weaving spider's spirally arrayed viscous capture thread (Figure 1a) remain compliant and stretch as they resist an insect's struggles to escape from the web (Figure 1b). As a thread's support line, which is composed of two flagelliform axial lines that pass through the center of each droplet's glycoprotein glue core (Figure 1e), bow under the tension generated by droplets that have adhered and extended, the adhesive forces of these droplets are summed in suspension bridge fashion (Figure 1c; Opell & Hendricks, 2007, 2009). This prey capture system is responsible for the success of the Araneoidea (Bond & Opell, 1998), a clade comprising 26% of all spider species and includes 17 families of orb‐weaving spiders and their descendants that spin webs with divergent architectures (Blackledge et al., 2009; Dimitrov et al., 2016; Hormiga & Griswold, 2014).

A growing number of studies on the properties and performance of orb‐weaver prey capture thread have revealed details about this natural adhesive system and how it responds to environmental humidity (Figure 1d), temperature, ultraviolet light, and insect surface texture (Opell Clouse & Andrews, 2018; Opell, Jain, et al., 2018; Opell & Schwend, 2007; Stellwagen, Opell, & Clouse, 2015, 2016; Stellwagen, Opell, & Short, 2014). Humidity has a pronounced effect on glue droplet adhesion, and interspecific differences in this response have been attributed to natural selection that optimizes thread performance to the humidity of each species' habitat (Amarpuri, Zhang, et al., 2015). This hypothesis was supported by a recent study of *Araneus marmoreus* Clerck, 1757, which determined that houseflies were retained 11 s longer at 72% relative humidity (RH) than at 37% or 55% RH by simple capture thread arrays. This is a very significant difference when a few additional seconds during the time required by a spider to locate, run to, and begin wrapping a prey with silk can mean the difference between a prey captured or a prey lost. In the current study, we extend this experimental approach to prey retention by threads from two additional large orb weavers, *Argiope aurantia* Lucas, 1833 and *Argiope trifasciata* (Forskål, 1775), which occupy different microhabitats (Brown, 1981).

Greater retention times should also translate into an ability to retain larger, more profitable prey, which comprises the largest proportion of an orb weaver's diet (Blackledge, 2011; Venner & Casas, 2005), but also see Eberhard (2013). The importance of large prey is also borne out in *A. trifasciata*. Most of this species' potential prey had body lengths <10 mm, whereas most prey taken had lengths >18 mm (Brown, 1981).

Capture thread adhesion has been characterized in two ways: (a) the force required to pull a thread or one of its glue droplets from a surface (Opell & Hendricks, 2009; Opell, Karinshak, & Sigler, 2013) and (b) the work done in pulling a thread or glue droplet from a surface (Opell, Clouse, & Andrews, 2018; Piorkowski & Blackledge, 2017; Sahni, Blackledge, & Dhinojwala, 2011). The latter approach probably best characterizes a thread's ability to overcome the work of an ensnared insect as it struggles to escape from a web. Many of the studies of capture threads have focused on the composition and performance of the glue droplets' outer hygroscopic aqueous layer and of its inner viscoelastic glycoprotein core. However, the elasticity of the thread's two supporting flagelliform axial fibers is also an important component of the suspension bridge mechanism (Blackledge & Hayashi, 2006a, 2006b; Opell & Hendricks, 2009; Opell, Markley, Hannum, & Hendricks, 2008; Sahni, 2010).

To better understand this highly integrated and compliant adhesive system, our study attempted to model the contributions glycoprotein adhesive and flagelliform fibers in the capture threads of *A. aurantia*, *A. trifasciata*, and *A. marmoreus*. Although the material properties of these three species' axial lines are reported in the literature (Sensenig, Agnarsson, & Blackledge, 2010), this required us to characterize the properties of their glycoproteins at each of the three experimental RH's (37%, 50%, and 72%) using recently developed techniques (Opell, Clouse, & Andrews (2018).

### 1.2 Viscous thread production, composition, and adhesion

A viscous capture thread is a self‐assembling adhesive system that forms when the products of a spider's paired posterior lateral spinnerets merge. A flagelliform spigot on each spinneret spins a protein fiber, which is coated with aggregate gland solution from two flanking spigots as it emerges (Coddington, 1989). The aggregate cylinder contains amorphous proteins as well as organic and inorganic low molecular mass compounds (LMMCs; Jain, Amarpuri, Fitch, Blackledge, & Dhinojwala, 2018; Townley & Tillinghast, 2013). This cylinder is quickly formed into a regular series of droplets by Plateau–Rayleigh instability (Edmonds & Vollrath, 1992; Roe, 1975). Within each droplet, a glycoprotein core coalesces and the remaining solution forms an aqueous layer, which covers both the glycoprotein core and flagelliform fibers, both within and between droplets, hydrating these components and maintaining their plasticity (Figure 1e). Other proteins that are not visible in microscopic examination remain in the aqueous layer (Amarpuri, Chaurasia, Jain, Blackledge, & Dhinojwala, 2015). Thus, our reference to and measurement of a droplet's glycoprotein or glycoprotein volume describe only the proteinaceous material that can be visualized with standard light microscopy.

The LMMCs serve several important functions. Along with glycoproteins, they confer hygroscopicity, causing the droplets' size and performance to change over the course of a day as they track environmental humidity (Figure 1d; Jain et al., 2018; Opell, Clouse, & Andrews, 2018; Opell, Jain, et al., 2018), they maintain glycoprotein structure and solvate glycoprotein, enhancing its surface interaction (Sahni et al., 2014), and they remove interfacial water from a droplet's contact footprint, enhancing adhesion (Singla, Amarpuri, Dhopatkar, Blackledge, & Dhinojwala, 2018). Optimal adhesion of an araneoid glue droplet is achieved when the viscosity of the droplet's glycoprotein is low enough to spread on a surface to establish sufficient adhesive contact, but high enough to ensure that the glycoprotein will cohere as it extends, thereby transferring adhesive force to the thread's axial lines (Figure 1c; Amarpuri, Zhang, Blackledge, & Dhinojwala, 2017; Amarpuri, Zhang, et al., 2015). Glycoprotein viscosity is determined primarily by the LMMCs in the droplets aqueous layer (Jain et al., 2018; Opell, Clouse, & Andrews, 2018; Opell, Jain, et al., 2018) and tuned to the humidity of an orb‐weaving species' habitat during a spider's foraging time (Amarpuri, Zhang, et al., 2015).

### 1.3 Microhabitat differences in study species

The previously studied species, *Araneus marmoreus*, builds webs in forest edge vegetation, where humidity does not drop greatly during late morning and afternoon (Opell, Buccella, Godwin, Rivas, & Hendricks, 2017). In the current study, we examined the effect of humidity on housefly retention by threads of the sympatric species *A. aurantia* and *A. trifasciata*, large orb weavers that construct webs in exposed, grassy and weedy habitats and are widely distributed in United States (Levi, 2004). East of the Mississippi River and along the Pacific coast *A. aurantia* and *A. trifasciata* are sympatric and can often be found in close proximity, where, to most observers, there is little to distinguish them ecologically. However, a study of Iowa and Indiana populations revealed microhabitat and prey differences between these species (Brown, 1981). *Argiope aurantia* is larger than *A. trifasciata* and constructs larger orb webs that have greater insect‐stopping power (Table 1). *Argiope aurantia* more commonly foraged in wetter, more herbaceous, later successional sites, whereas *A. trifasciata* was more common in dryer, grassy, early successional habitats, where it built webs an average of 6.8 cm higher in the vegetation than did *A. aurantia* (Brown, 1981). These differences resulted in *A. aurantia* capturing a greater proportion of jumping prey and *A. trifasciata* a greater proportion of flying prey, with prey captured by *A. aurantia* having a 22% greater body length than those captured by *A. trifasciata* (Table 1). The capture of larger prey by *A. aurantia* is consistent with the greater adhesiveness of their capture threads (Table 1). The extensibility per glycoprotein volume of *A. aurantia* droplets peaks at 55% RH and then decreases (Opell et al., 2013), suggesting that its threads will retain flies longer at 55% RH. However, this index has not been determined for *A. trifasciata*.

**Table 1:** Comparison of the size, web features, capture thread properties, and prey size of *Argiope aurantia* and *A. trifasciata*. Values from Brown (1981) are means of those reported for several populations of each species. Adhesion from Opell, Lipkey, et al. (2009) is that of outer capture spirals from early season webs

	*Argiope aurantia*	*Argiope trifasciata*	Reverence
Spider size			
Carapace width (mm)	4.7 ± 0.60	3.5 ± 0.62	Sensenig et al. (2010)
Cephalothorax‐abdomen length (mm)	20.1 ± 0.7	16.7 ± 0.6	Brown (1981)
Mass (mg)	392 ± 278	133 ± 89	Sensenig et al. (2010)
Web features			
Web height above ground (cm)	52.5 ± 2.8	60.8 ± 1.5	Brown (1981)
Web radius (cm)	16.0 ± 1.1	15.8 ± 3.3	Brown (1981)
Web capture area (cm^2^)	438 ± 215	448 ± 275	Sensenig et al. (2010)
Stopping power (µJ cm^2^)	60 ± 26	39 ± 24	Sensenig et al. (2010)
Capture threads			
Spiral spacing (mm)	4.8 ± 0.7	3.3 ± 1.2	Sensenig et al. (2010)
Droplets per mm	3.5 ± 0.4	6.1 ± 0.6	Opell and Hendricks (2009)
Adhesion (µN/2,133 µm)	330 ± 30	230 ± 25	Opell, Lipkey, et al. (2009)
Adhesion per area (µN/cm^2^)	190 ± 155	89 ± 77	Sensenig et al. (2010)
Prey size			
Body length of prey (mm)	11.22 ± 1.98	9.23 ± 1.48	Brown (1981)

Although in the United States *A. aurant*ia and *A. trifasciata* are commonly found in close proximity, they are not closely related (Cheng, Yang, Lin, Herberstein, & Tso, 2010). *Argiope aurantia*, which is confined to North America, is more closely related to species from Africa and Eurasia. *Argiope trifasciat*a is also found in Eurasia and Africa, but has a closer affinity with species from Asia. In Europe *A. trifasciata* is found in warmer, more arid habitats (Di Pompeo, Kulczycki, Legittimo, & Simeon, 2011), consistent with the observations of Brown (1981). Thus, it is likely that *A. trifasciata* has moved into the range of *A. aurantia*.

## 2 METHODS AND MATERIALS

### 2.1 Species studied and procedures for collecting their threads

For both fly retention tests and viscous droplet characterization, we collected sectors from orb webs constructed by adult female *A. aurantia*, *A. trifasciata*, and *A. marmoreus* living near Blacksburg, Virginia. These webs were captured on 15 × 52 cm rectangular aluminum frames with double‐sided tape (Cat. # 9086K29550360, 3M Co., Maplewood, MN, USA) applied to their 1 cm faces to maintain native thread tension. Frames were transported and kept in closed boxes to prevent threads from being contaminated by dust and pollen and were stored in the laboratory at 50% RH prior to use. The web samples of different spiders were used in fly retention and droplet characterization studies, although each study used spiders from the same local populations. In each case, we collected thread samples between 06:00 and 09:00 and completed their study by 16:00 on the day of collecting, ensuring that thread age did not impact our results. We collected threads used in the current fly retention tests from the webs of 20 adult *A. aurantia* females and 21 adult *A. trifasciata* females between 16 August and 19 October 2016. Thread samples used to characterize droplets were collected from 14 adult *A. trifasciata* and 14 adult *A. aurantia* between 9 September and 3 October 2014 and between 29 August and 30 September 2011, respectively.

In the laboratory, we isolated the capture thread spans in an inter‐radius web sector by placed 5‐mm‐wide brass bars covered on their lower surfaces with double‐sided carbon tape (Cat #77816; Electron Microscope Sciences, Hatfield, PA, USA) across the width of the collecting frame and along adjacent web radii. This secured the radial threads to the tape's adhesive and allowed us to remove capture threads without altering the tension of threads in adjacent web sectors. We used tweezers whose tips were covered in double‐sided carbon tape and blocked open to accommodate the spacing of supports used for insect retention trials or of supports on microscope slides used for droplet characterization. A hot wire probe was used to sever each end of a thread before it was removed from the web sample to avoid stressing the thread and to ensure that its naïve in‐web tension was maintained.

### 2.2 Assessing insect retention times

Translating these humidity‐mediated material properties of orb spider glycoprotein glue into prey retention performance is challenging because many factors other than capture thread adhesion affect prey retention by orb webs. These include web orientation (horizontal vs. vertical), velocity of insect impact, the number of capture spirals contacted, the surface texture of the insect body regions that contact capture threads, the insect's struggle behavior, and whether or not an insect readheres to capture threads after pulling free of one set of threads and tumbling into lower capture thread spirals of a vertical orb web (Blackledge & Zevenbergen, 2006; Opell & Schwend, 2007; Zschokke & Nakata, 2015). As in the previous study of *A. marmoreus* (Opell et al., 2017), we attempted to control for as many of these variables as possible by preparing three horizontally oriented capture thread arrays from each spider's web sample and by placing an anesthetized adult *Musca domestica* Linnaeus, 1758 housefly wings downward on the center of each array.

**Figure 2:**
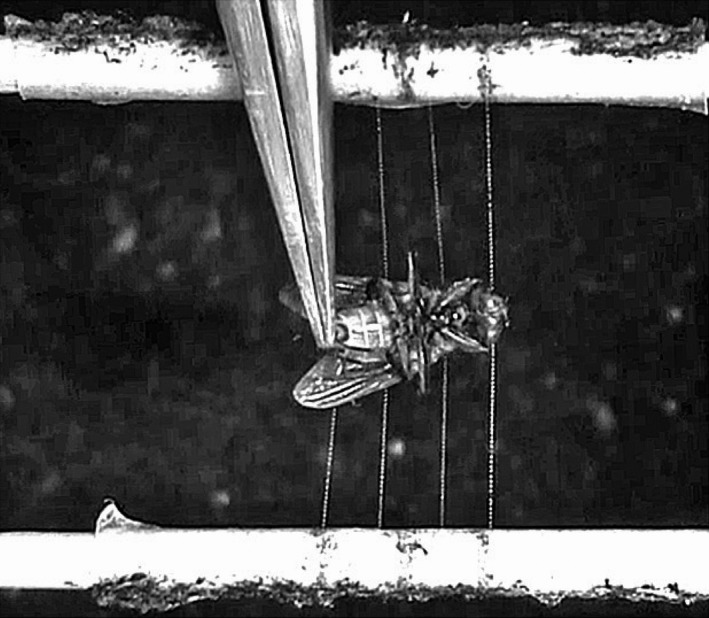
Screen capture from a fly retention video recording, showing a CO_2_ anesthetized housefly being placed onto four 16‐mm‐long capture thread strands, spaced at 2‐mm intervals between parallel wooden applicator stick supports

Each thread array consisted of four equally spaced capture threads suspended across the 16 mm space separating two 2.5‐mm‐diameter wooden applicator sticks mounted in parallel across a ring support (Figure 2). Double‐sided 3M tape covered central region of each applicator stick support to ensure thread adhesion. When we initially attempted to use three capture threads spaced at 3‐mm intervals, as was done in the study of *A. marmoreus* prey retention, we found that flies escaped too quick from *Argiope* threads to provide the retention time resolution that we desired. Therefore, we used four threads spaced at 2‐mm intervals, maintaining the same 6 mm spacing between the two outermost threads (Figure 2).

Plexiglas desiccator cabinets again served as chambers where 37%, 55%, and 72% RH was established (Table 2), being monitored with a digital hygrometer (model 11‐661‐7A, Thermo Fisher Scientific, Waltham, MA, USA). We maintained 37% RH by a using a small Peltier‐based dehumidifier (model 236072, Ivation, Boise, ID, USA) and dishes of silica gel desiccant. Because ambient room humidity was about 50% RH, a distilled‐water‐moistened paper towel and gentle exhaling into the 55% RH chamber were sufficient to maintain this humidity. In the third chamber, a small ultrasonic humidifier (USB, 160 ml Volcano model, Foxwill, Inc., Rosemead, CA, USA) and 4‐cm square electronic cooling fan established 72% RH.

**Table 2:** Experimental conditions of study

Nominal % RH	*Argiope aurantia* (*N* = 20)			*Argiope trifasciata* (*N* = 21)		
	RH	Temp °C	Absolute humidity	RH	Temp °C	Absolute humidity
37	38.3 ± 0.4	23.3 ± 0.2	8.00 ± 0.08	38.7 ± 0.42	23.1 ± 0.11	7.98 ± 0.09
55	54.7 ± 0.4	23.3 ± 0.2	11.45 ± 0.13	55.0 ± 0.23	23.0 ± 0.07	11.33 ± 0.06
72	71.8 ± 0.5	23.1 ± 0.1	14.85 ± 0.12	70.9 ± 0.60	22.9 ± 0.08	14.46 ± 0.15
*p* value	**W = .0001**	W = .2753	**A = .0001**	**W < .0001**	W = .2024	**W < .0001**

Note

Mean ± 1 standard error; A = ANOVA test; W = Wilcoxon test. Significant *p* values are in bold text.

We monitored temperature using a thermometer suspended 1 cm below the top of each chamber and maintained at 23°C to standardize our observations to both the previous insect retention study (Opell et al., 2017) and studies of capture thread droplet properties (Opell, Clouse, & Andrews, 2018; Opell et al., 2013; Stellwagen, Opell, & Clouse, 2015, 2016; Stellwagen et al., 2014). To 37% RH and 72% RH chambers, we added a 22 × 15 cm aluminum heat sink that extended 11 cm into the top of the chamber and served to dissipate the heat added by the dehumidifier and humidifier, respectively. We were also able to place an ice pack on these heat skinks as needed to maintain a temperature of 23°C (Table 2).

We assayed insect retention using adult *Musca domestica* houseflies, purchased as pupae from the same source as our previous study (item100002365, Evergreen growers Supply Oregon City, OR, USA) and kept at 14°C until being warming to 23°C in small groups to provide flies for testing. Each group of pupae was warmed at 4‐ to 5‐day intervals to provide vigorous flies for tests. Flies were provided with continuous access to distilled‐water‐saturated cotton and a small dish of granulated sugar. An hour before each day's tests, flies were placed individually in a clean, cotton stoppered glass vials. A four‐thread array was then placed into a chamber at least two minutes before a fly was placed on threads. This corresponds to the acclamation times used to characterize the effects of humidity on the volumes of viscous droplets and their glycoprotein cores and their material properties (Opell, Clouse, & Andrews, 2018; Opell et al., 2013). Each fly was used for only one trial, being lightly anesthetized for approximately 5 s with CO_2_ dispensed into its vial from a tank before being gently pressed into the threads, winds downward, so that its head contacted the first strand and its wings contacted the fourth strand (Figure 2). Each fly was centered on the thread array. Although care was taken in fly placement, differences in the force with which a fly was pressed against the thread array have the potential to affect glycoprotein spreading on the fly's setae and exoskeleton and, therefore, adhesion to prey.

A pooled sample of 30 adult flies had a mean mass of 12.03 mg per fly, very similar to the 12.01 mg mean fly mass of our previous study (Opell et al., 2017). A sample 10 flies from the current study were also similar in size to a sample of six flies from the previous study (values in parentheses): head width across eyes = 2,185 µm ± 40 µm standard error (2,220 µm ± 40 µm) and notum width = 2,116 µm ± 40 µm (2,110 µm ± 70 µm), neither measurement differing significantly (*t* test *p* = .4966 and .9650, respectively).

We recorded fly escape behavior at 60 frames per second using a Canon Vixia HF 610, HD camcorder that rested, lens downward, on the top of a humidity chamber, starting a video recording as the fly was being placed on a thread array and stopping the recording shortly after a fly escape occurred. An escape was considered to have occurred when a fly either completely escaped the thread array (usually by dropping to the bottom of the chamber) or contacted an applicator stick so that it was able to begin pulling itself free from the threads and subsequent behavior differed from what would have occurred in an orb web. We recorded humidity at the beginning of fly struggle and again at the time of fly escape, as defined above, using the mean of these two values to characterize the trial's humidity. Temperature was recorded when a fly began to struggle.

We reviewed each video with iMovie® (version 10.1.4), recording the length and number of active struggle bouts prior to escape from a thread array. Each activity bout was characterized as either leg struggle or wing flap, which could also include leg movements. It appeared that wing flapping was the more energetic of the two behaviors and had the greatest potential to facilitate escape. Thus, a fly's behavior was summarized as total number of activity bouts, total leg struggle time, total wing flap time, and total active struggle time. To account for interindividual variance in capture thread adhesion, we also followed the approach used in our previous study (Opell et al., 2017), computing the mean leg struggle, wing flap time, and total active struggle time for the three humidities for each individual and then, for each humidity, determining the deviation of each index from its respective mean value. Thus, a retention time less than meantime would have a negative value and one greater than the mean a positive value.

As noted, with one exception, the experimental procedures of this study were identical to those of our previous study (Opell et al., 2017). Thread arrays of *A. marmoreus* contained three strands, whereas those of *A. aurantia* and *A. trifasciata* each contained four strands. To account for this difference in prey retention modeling, we multiplied each *A. marmoreus* individual's total active struggle time by 4/3 before developing a common model. Given the same 6 mm spacing between the outer threads of the arrays used in both studies, we believe that this scaling brought the two studies' retention times into line.

### 2.3 Characterizing droplet performance features

**Figure 3:**
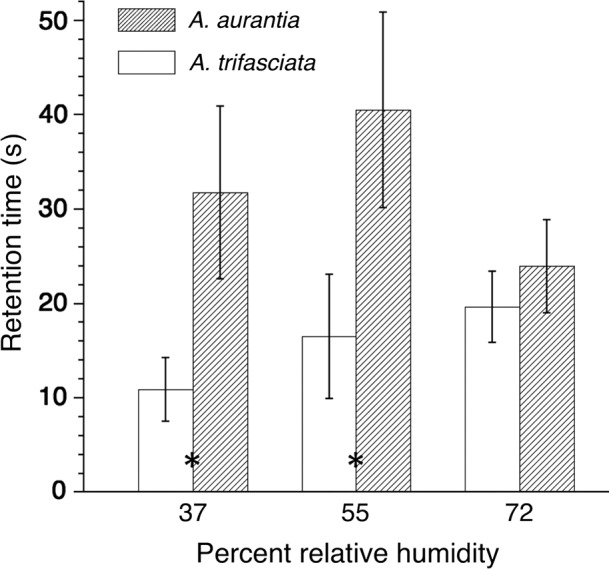
Comparison of housefly retention times of *Argiope trifasciata* and *A. aurantia* by capture thread arrays (mean ± 1 standard error. Wilcoxon *p*: *A. trifasciata* = .0366, *A. aurantia* = .7950). Stars indicate interspecific differences between retention times (Wilcoxon 2‐sample normal approximation *p*: 37% RH = .0068, 55% RH = .0127, 72% RH = .5749)

We transferred capture threads to samplers made of U‐shaped brass supports epoxied at 4.8‐mm intervals to microscope slides with their free ends extending upward and covered with double‐sided carbon tape (Figure 3 in Opell, Tran, and Karinshak, 2011). Two sets of thread samples were prepared from each web, one used to measure droplet volume and glycoprotein contact surface area and the other used to characterize droplet extension. Each set of measurements was made within a temperature and humidity‐controlled chamber that rested on the mechanical stage of a Mitutoyo FS60 inspection microscope (Mitutoyo America Corp., Aurora, IL, USA), where a temperature of 23° and RH's of 20%, 37%, 55%, 72%, and 90% were established (Opell et al., 2013).

We determined glycoprotein contact areas from images of three suspended droplets that were subsequently flattened to reveal their glycoprotein cores and then rephotographed. Flattening was achieved by dropping a glass coverslip onto them from a release mechanism contained within the humidity‐controlled observation chamber (Opell et al., 2013). Using ImageJ (Rasband, 1997–2012), we measure the length (DL; dimension parallel to the axial fiber) and width (DW) of suspended droplets and the surface areas of flattened droplets and of their glycoprotein cores. Droplet volume (DV) was determined using the following formula (Liao, Blamires, Hendricks, & Opell, 2015; Opell & Schwend, 2007).

(1) $$\text{DV}=\frac{2Pi\times {\text{DW}}^{2}\times \text{DL}}{15}$$

We computed the volume of a droplets consolidated glycoprotein core at each humidity by first dividing a droplet's volume by its flattened area to determine its thickness. Droplet thickness was equated with glycoprotein thickness (Opell et al., 2013) and then multiplied by flattened glycoprotein core surface area to determine glycoprotein volume. For each droplet, the ratio of the glycoprotein core volume to droplet volume was determined. For each individual, we determined mean glycoprotein core volume to droplet volume ratio at each humidity. These ratios were then multiplied by the volumes of this individual's droplets that were extended to infer the volume of glycoprotein core within the droplet that was extended.

To ensure that the probe used to contact and extend a droplet contacted only a single droplet, we used a minute insect pin or the finely pointed tip of a wooden applicator stick moistened with distilled water to slide adjacent droplets away from the indented test droplet, which was located at the center of the thread strand. A dissecting microscope allowed us to position the small bundle of xylem fibers extended from the applicator's tip near the droplet to be moved such that the droplet designated for extension was not disturbed. This process retained the aqueous coating of the strand's axial fibers, as demonstrated by the formation of small droplets similar to those often present between the large primary droplets of many viscous threads.

To confirm that sliding droplets away from the isolated droplet to be extended did not withdraw material from this droplet, we used matched paired tests to compare the volume of an individual's extended droplets with then mean value of its suspended droplets that were subsequently flattened to reveal their glycoprotein cores (Table 3). The only significant difference identified was in *A. aurantia* at 72% RH, where isolated droplet volume exceeded that of native droplet volume. Thus, there is no evidence that preparing droplets for extension altered their properties.

**Table 3:** Comparisons between native and isolated thread droplet volumes

	37% RH		55% RH		72% RH	
	Native	Isolated	Native	Isolated	Native	Isolated
*Argiope aurantia*	65,146 ± 10,065	82,082 ± 16,258	71,139 ± 12,686	86,126 ± 16,474	78,806 ± 17,186	106,789 ± 23,062
*p* value	.0876		.1793		.0427	
*A. trifasciata*	37,236 ± 6,797	43,740 ± 8,123	44,414 ± 9,184	41,585 ± 6,896	44,998 ± 9,667	52,556 ± 9,065
*p* value	.5777		.2783		.6932	
*A. marmoreus*	92,548 ± 16,268	91,310 ± 10,699	81,329 ± 12,601	96,961 ± 12,100	114,757 ± 16,270	108,270 ± 15,872
*p* value	.6251		.0824		.5092	

Note

Mean ± 1 standard error and two‐tailed matched‐pairs *t* test *p* value.

We photographed the isolated droplet before it was extended and used this image to infer its glycoprotein volume as described above. A steel probe, cleaned with 100% ethanol on a Kimwipe® before each use, was inserted through a port in the side of the test chamber and its 413‐μm‐wide polished tip aligned and brought into contact with the focal droplet. The probe was anchored, and then, to ensure full droplet adhesion, its tip pressed against the droplet until its support line was deflected by 500 μm. A 60‐fps video recorded the droplet's extension as the thread was withdrawn from the probe at a velocity of 69.6 μm/s by a stepping motor that advanced the mechanical stage on which the observation chamber rested. These videos allowed us to measure the maximum length of the extended droplet filament at pull‐off.

### 2.4 Characterizing thread material properties

The diameter, Young's modulus, and toughness of *A. aurantia*, *A. trifasciata*, and *A. marmoreus* flagelliform axial lines were taken from the literature (Table S1; Sensenig et al., 2010). We multiplied the total cross sectional area (CSA) of each species' paired axial lines by 1 m, obtaining their volume in m^3^, and then multiplied this value by it axial line toughness to determine the work of extending these fibers by 1 m. Although the capture threads clearly did not extend this far during fly struggle, this index allowed us to evaluate the contribution of axial line work of extension to prey retention.

Using recently developed techniques (Opell, Clouse, & Andrews, 2018), we characterized the toughness of *A. aurantia*, *A. trifasciata*, and *A. marmoreus* glycoproteins at each of the three experimental humidities. We performed this analysis for what this previous study termed Phase 1 extension, the first portion of a droplet's extension during which the extending glycoprotein filament is completely covered by aqueous material and has not transitioned to Phase 2, where small droplets of aqueous material form on the filament, exposing portions of the glycoprotein. Phase 1 corresponds to droplet performance typically observed in the course of thread pull‐off (Opell, Clouse, & Andrews, 2018). The droplets whose extensions were characterized are the same ones whose extensions are described above.

As the details of this method are explained in the literature (Opell, Clouse, & Andrews, 2018), we review only the basic procedures here. At the first indication of droplet extension, we measured deflection angle of the thread's support line and assigned a droplet length equal to the diameter of the droplet's glycoprotein core when configured as a sphere. At subsequent 20% extension intervals, we measured both filament length and support line angular deflection. This increase the resolution of the procedure, compared with the referenced study, which used 25% extension intervals. We computed true strain at each 20% extension interval as the natural log of the droplet filament's length divided by the diameter of its glycoprotein core when configured as a sphere. We computed the true stress on a filament at the six extension intervals by dividing the force on the filament by its CSA. At the initiation of extension, CSA was computed as that of a cylinder with a height equal to the diameter of a glycoprotein core when configured as a sphere. At the remaining five intervals, CSA was determined by dividing glycoprotein volume by filament length. Knowing that each support line had an initial length of 4,800 µm with the extended droplet situated at its center and knowing the diameters and Young's modulus of each species' axial lines (Table S1; Sensenig et al., 2010), we used the angular deflection of the support line to calculate the force that each side of the line exerted on the droplet. Support line deflection was then used to resolve the force vectors of both sides of the support lines into the force on the extending glycoprotein filament.

### 2.5 Modeling contributions of flagelliform fibers and glue droplets to prey retention time

We used a minimum BIC forward selection stepwise regression model, implemented with JMP (SAS Institute, Cary, NC, USA) to evaluate the contributions of capture thread features to total active struggle time, which we also term “retention time” and express in seconds. Axial line features included the following: CSA, Young modulus, and toughness, all taken from the literature (Table S1; Sensenig et al., 2010), plus the work of extending axial lines, as described above. Droplet properties included the following: droplets per mm thread length, flattened droplet area, and the volume, flattened area, and thickness of glycoprotein in each droplet and their values per mm thread length. We also included glycoprotein flattened area per volume and droplet extension and extension per glycoprotein volume. Glycoprotein material properties included Young's modulus and toughness. Multiplying glycoprotein toughness by a droplet's glycoprotein volume yielded the work of extending a droplet, which we multiplied by droplets per mm to obtain the work of pulling free the droplets of a mm length of thread.

The hygroscopic aqueous layer that surrounds a droplet's glycoprotein core also surrounds axial lines in interdroplet thread regions (Figure 1a,e). Therefore, we expect that as the water content of the aqueous layer increases with humidity axial line material properties change slightly as they become more hydrated. To approximate this change, we computed adjusted axial line Young's modulus, toughness, and work of extension values for 37% and 72% RH conditions to reflect the fact that values reported in the literature were measured at about 50% RH. We adjusted 37% RH Young's modulus values by increasing 55% RH values by 10% and adjusted 72% RH values by decreasing 55% RH values by 10%. Our characterization of glycoprotein properties showed that as humidity increased Young's modulus and toughness of *A. marmoreus*, *A. trifasciata*, and *A. marmoreus* glycoproteins decreased (Table S1). We also found that across humidities within each species glycoprotein Young's modulus and toughness were positively correlated (*r* = .73, .97, and .89, respectively, *p* < .0001 in all species). Therefore, we also increased 55% RH toughness and work of extension by 10% to derive adjusted 37% RH values and reduced 55% RH values by 10% to derive adjusted 72% RH toughness and work of extension values.

### 2.6 Statistical analysis

We used JMP to analyze data, considering comparisons with *p* ≤ .05 as significant. All matched pair tests were two‐tailed. The normal distributions of values were assessed, with a Shapiro–Wilk W test, with values of *p* > .05 being considered normal. We used parametric statistics to compare variables when the values of all treatments were normally distributed and nonparametric statistics when the values of one or more treatments were not normally distributed. In minimum BIC forward selection stepwise regression modeling, only variables that made significant contributions, as judged by *p* values <.05 and LogWorth (LW) and False Discovery Rate LogWorth (FDLW) values >2.0, were retained in the regression model.

## 3 RESULTS

### 3.1 Prey retention time comparisons

The behavior that immediately proceeded escape from a thread array was similar in both species (Table 4): leaving without contacting an applicator stick, often at the end of a wing flap bout (62%), contacting an applicator stick with one or more legs and being in a position to begin pulling from threads (35%), and simply dropping from a thread array without notable struggle at the time (3%). Humidity did not affect wing flap, leg struggle, or total struggle times required by flies to escape from *A. aurantia* thread arrays or the deviations from the mean values of each of these three indices (Tables 5 and 6). However, all values of this species were the greatest at 55% RH (Table 5, Figure 3). The total struggle time that flies required to escape from *A. trifasciata* threads increased with humidity (Table 5, Figure 3). Neither leg struggle nor wing flap differed among humidity treatments. However, the deviation of *A. trifasciata* mean wing flap and mean total struggle time increased with humidity (Table 6).

**Table 4:** Frequency of behavior that immediately proceeded fly escape from a thread array. Fall denotes a fly that simply fell from the threads without notable struggle; Contact, a fly that “escaped” by contacting an applicator stick and, therefore, was capable of pulling itself from the threads; and Leave, a fly that freed itself from the web by active struggle without contacting an applicator stick

% Relative humidity	*Argiope aurantia* (*N* = 20)			*Argiope trifasciata* (*N* = 21)		
	Fall	Contact	Leave	Fall	Contact	Leave
37%	0	7	13	1	7	13
55%	1	7	12	0	8	13
72%	1	5	14	1	9	11

**Table 5:** Housefly retention times. Time spent in wing flapping and leg struggle escape behaviors as well as total struggle time and the number of activity bouts in each fly escape episode

Nominal % RH	*Argiope aurantia* (*N* = 20), s				*Argiope trifasciata* (*N* = 21), s			
	Wing flap	Leg struggle	Total time	Activity bouts	Wing flap	Leg struggle	Total time	Activity bouts
37	2.05 ± 0.85	29.97 ± 9.22	32.02 ± 9.58	11.8 ± 3.91	1.96 ± 0.73	8.90 ± 3.41	10.85 ± 3.37	4.7 ± 1.26
55	4.21 ± 2.75	36.30 ± 9.64	40.50 ± 10.35	12.1 ± 3.16	2.52 ± 0.75	13.99 ± 6.61	16.51 ± 6.60	4.3 ± 1.34
72	2.30 ± 0.81	21.64 ± 5.20	23.94 ± 4.96	10.2 ± 2.01	7.18 ± 2.25	12.48 ± 2.69	19.65 ± 3.79	4.7 ± 0.78
*p* value	W = .6581	W = .5093	W = .7629	W = .6528	W = .2239	W = .1752	W = **.0366**	W = .2957

Note

Mean ± 1 standard error; A = ANOVA test; W = Wilcoxon test. Significant *p* values are in bold text.

**Table 6:** Deviations from an individual's mean total fly retention time and wing flap and leg struggle components. Mean ± 1 standard error. A = ANOVA test. W = Wilcoxon test

Nominal % RH	*Argiope aurantia* (*N* = 20), s			*Argiope trifasciata* (*N* = 21), s		
	Wing Flap	Leg Struggle	Total Time	Wing Flap	Leg Struggle	Total Time
37	−0.80 ± 1.22	0.67 ± 7.07	−0.134 ± 7.21	−1.93 ± 1.05	−2.89 ± 3.44	−4.82 ± 3.63
55	1.35 ± 1.85	6.99 ± 7.10	8.346 ± 7.52	−1.36 ± 0.82	2.20 ± 4.05	0.84 ± 4.26
72	−0.55 ± 0.86	−7.66 ± 5.32	−8.21 ± 5.28	3.29 ± 1.57	0.69 ± 2.22	3.98 ± 3.16
*p* value	W = .871	W = .710	W = .688	**W = .0251**	W = .2265	**W = .0349**

Note

Mean ± 1 standard error; A = ANOVA test; W = Wilcoxon test. Significant *p* values are in bold text.

The total retention times of *A. aurantia* exceeded those of *A. trifasciata* at 37% and 55% RH, but not at 72% RH (Figure 3). The similarity at 72% reflected a decrease in *A. aurantia* time at this humidity. The number of activity bouts was not affected by humidity in either species (Table 5). However, when the three humidity treatments were combined, each species exhibited a positive relationship between the number of activity bouts and total struggle time (*A. aurantia,* Total Time = 2.58 Bouts + 2.84, *p* < .0001, *R* ^2^ = .85; *A. trifasciata,* Total Time = 2.44 Bouts + 4.48, *p* < .0001, *R* ^2^ = .33). Thus, the more securely a fly was held by a thread array, the more activity bouts it required to escape.

### 3.2 Effect of humidity on droplet features

**Figure 4:**
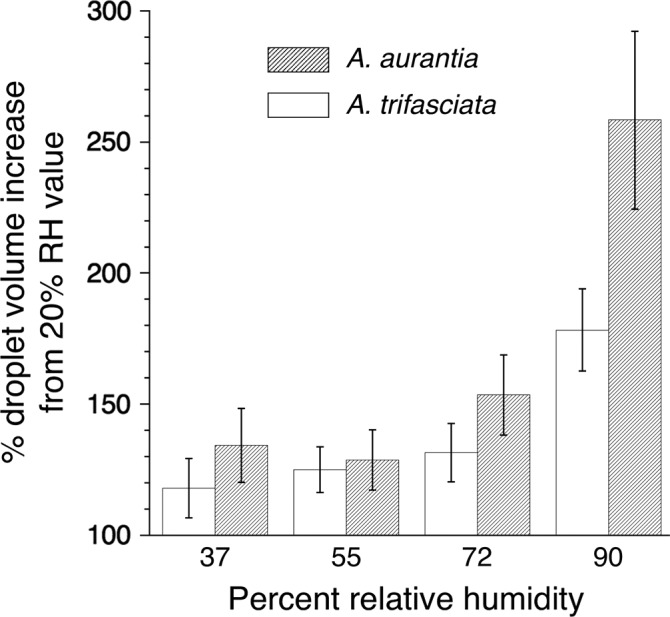
Comparison of mean *Argiope trifasciata* and *A. aurantia* viscous droplet hygroscopicity at four humidities expressed as a percent of the value observed for each individual at 20% RH. Sample size: *A. trifasciata* = 14, *A. aurantia* = 13, error bars ± 1 standard error. Values at each RH were normally distributed for each species, and analysis of variance tests were significant for each species (*p* < .0001)

**Figure 5:**
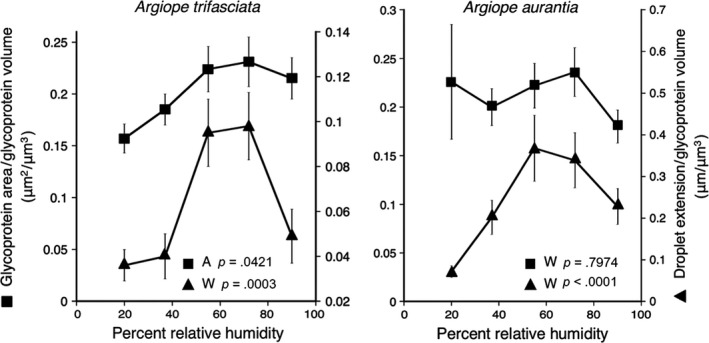
Effects of humidity on droplet extension per glycoprotein volume and glycoprotein surface area per glycoprotein volume of *Argiope trifasciata* and *A. aurantia* (mean ± 1 standard error). These differences suggest that *A. trifasciata* thread performance should peak at 72% relative humidity (RH), and, owing to a decrease in droplet extension at 72% RH, that *A. aurantia* threads should perform best at 55% RH

The effects of relative humidity on the two *Argiope* species' droplet features are reported in Tables S2 and S3. Contrary to expectations from microhabitat differences (Table 1), the droplets of *A. aurantia* and not *A. trifasciata* were more hygroscopic (Figure 4). However, the impact of humidity on each species' glycoprotein surface area and extension (each expressed relative to glycoprotein volume; Figure 5) corresponds to its prey retention performance (Figure 3). In *A. trifasciata,* both values increased up to 72% RH and then decreased at 90% RH, corresponding to a progressive increase in this species' prey retention time from 37% to 72% RH. In contrast, *A. aurantia* surface area increased from 37% to 72% RH, but after increasing from 20% to 55% RH, its droplet extension decreased at 72% RH. These associations suggest that glycoprotein extension makes a greater contribution to prey retention than does glycoprotein flattened area. In both species, reduced glycoprotein extension appears to occur when glycoprotein becomes overlubricated at higher humidities and its cohesion drops (Amarpuri, Zhang, et al., 2015; Opell et al., 2013; Sahni et al., 2011). The greater hygroscopicity of *A. aurantia* droplets (Figure 4) explains why this species' threads start to become overlubricated at lower humidities than those of *A. trifasciata*.

### 3.3 Glycoprotein material properties

**Figure 6:**
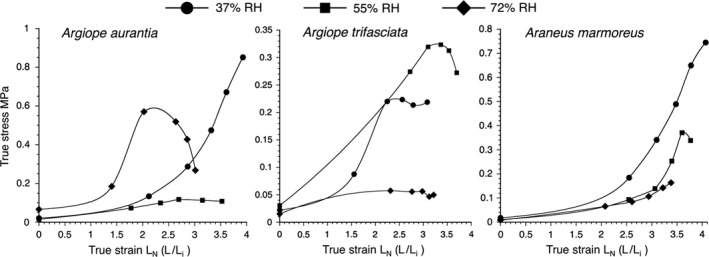
Stress–strain curves for *Argiope aurantia, A. trifasciata,* and *A. marmoreus* glycoproteins at three test humidities. Values used in computing these curves are reported in Tables S5–S7

The features of *A. aurantia*, *A. trifasciata*, and *A. marmoreus* glue droplets are reported in Tables S1–S4, the measurements used to determine their Young's modulus and toughness values in Tables S5–S7. The three species' stress–strain curves are shown in Figure 6.

### 3.4 Linking droplet properties and retention time

The regression model of insect retention time (active struggle time) in seconds (IRS) with the smallest overall *p* value (<.0001), smallest mean component *p* value (three components ≤.0001, other two components .0004 and .0016), and the largest mean LW and FDRLW values (range 2.789–4.453 and 2.789–3.904, respectively) included five variables: glue droplets per mm (DPMM), adjusted axial line work per meter length in nJoule (ALW), glycoprotein volume per droplet in µm^3^ (GV, taken from “glycol area and volume” values), glycoprotein thickness in µm (GT), and glycoprotein Young's modulus in MPa (GYM).

(2) $$\begin{array}{c} \text{IRS}=-3.3649\;\text{DPMM}+0.0203\;\text{ALW}+0.0016\;\text{GV}\\ +11.2739\;\text{GT}-63.9969\;\text{GYM}-91.6738. \end{array}$$

Both axial line work and adjusted axial line work were positively related to retention time (*R* ^2^ = .59 and .55, respectively, *p* = .0162 and .0214, respectively). No other model variable was individually related to retention time. Other regression models had lower fitnesses and included more variables, which introduced redundant contributions of flagelliform and glycoprotein features.

**Figure 7:**
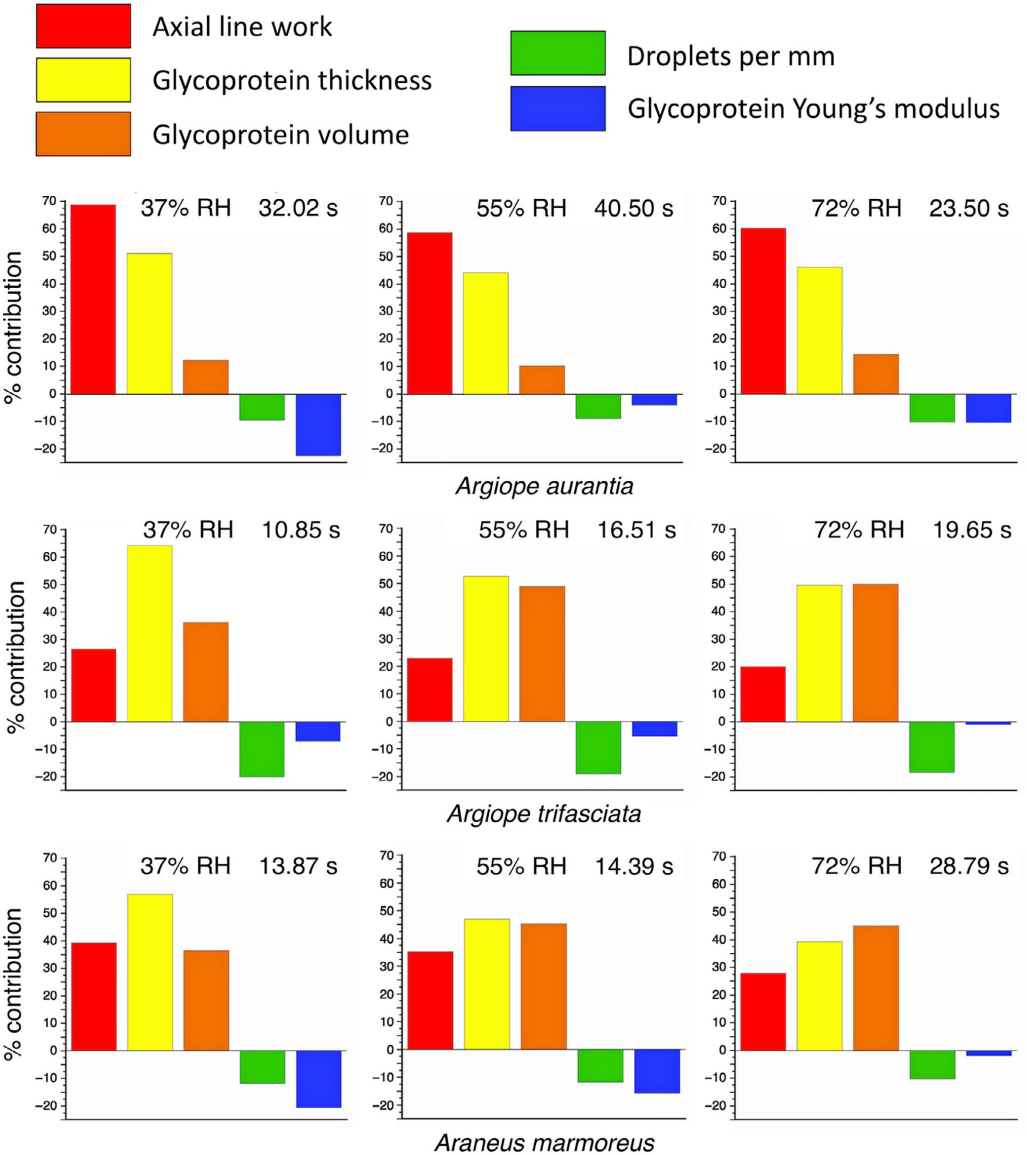
Modeled contributions of five thread properties to the total retention times in seconds of *Argiope aurantia, A. trifasciata,* and *A. marmoreus* thread arrays. Retention seconds (s) are shown at the upper right of each histogram

Although robust, this is a statistical model and not an engineering formulation. It is also unique to these three species and three humidities. However, as shown in Figure 7, the model does document that both axial line and glycoprotein properties contribute to a capture thread's ability to retain prey and that the nature of these contributions differs among species and humidities.

Axial line work reflects the contribution that flagelliform fibers make to the work that an insect must do to escape from threads. Its greater consistent contribution in *A. aurantia* reflects both the greater toughness of this species' flagelliform fibers and their greater diameters (Table S1). Although both glycoprotein thickness and volume were included in the model, their values were not related (*r* = .43, *p* = .2457). Glycoprotein thickness may account for several things: the effect of humidity on glycoprotein viscosity, the ability of glycoprotein to spread on an insect surface, and its ability to surround and interact with the setae on an insect's surface (Opell & Schwend, 2007). In contrast, glycoprotein volume appears to serve principally to account for the similarity of *A. trifasciata* and *A. marmoreus* glycoprotein volumes, which are much larger than those of *A. aurantia* (Tables S2 and S3). Likewise, DPMM appears to distinguish *A. trifasciata* with 6.1 DPMM from *A. aurantia* with 3.5 DPMM and *A. marmoreus* with 3.7 DPMM (Opell & Hendricks, 2009). The negative contribution of glycoprotein Young's modulus indicates that prey retention is favored by glycoprotein that more easily extends. Thus, as humidity increases and glycoprotein Young's modulus decreases, it detracts less from prey retention time.

When model component contributions are compared (Figure 7), the relative contributions of axial line work, glycoprotein thickness, and glycoprotein volume of *A. trifasciata* are more similar to those of *A. marmoreus* than to those of *A. aurantia*, with glycoprotein thickness and glycoprotein volume tending to dominate in the first two species. In contrast, in *A. aurantia* axial line work dominates with glycoprotein thickness making a strong contribution, but glycoprotein volume only a minor contribution. The differences in *A. aurantia* and *A. trifasciata* axial line contributions result not from a difference in the toughness of their flagelliform fibers, but from the greater diameters of *A. aurantia* flagelliform fibers (Table S1). Likewise, although the toughnesses of *A. aurantia* and *A. trifasciata* glycoproteins are similar, the volumes of glycoprotein within the droplets of *A. trifasciata* and *A. marmoreus* are more similar (Tables S2–S4).

## 4 DISCUSSION

*Argiope aurantia* and *A. trifasciata* are found in different microhabitats (Table 1) and their viscous prey capture threads had different properties (Tables S1–S4), exhibited different responses to humidity (Figures 4 and 5), and had different prey retention characteristics (Figure 3). *Argiope aurantia*, the larger of the two species, spins capture threads with larger, more widely spaced droplets, but with a smaller glycoprotein core within each droplet. *Argiope aurantia* droplets were more hygroscopic, causing their glycoproteins to become oversaturated at humidities greater than about 55% RH and their thread's prey retention time to drop thereafter (Figure 3). In contrast, *A. trifasciata*, whose capture threads exhibited shorter retention times at 37% and 55% RH than those of *A. aurantia*, showed a continual increase in retention as humidity increased, approximating the retehtion time of *A. aurantia* at 72% RH.

A model that combined viscous capture thread glycoprotein and flagelliform fiber properties confirmed that both components make important contributions to capture thread adhesion (Opell & Hendricks, 2007, 2009; Opell, Markley, et al., 2008; Sahni et al., 2010; Sahni et al., 2011) and illustrated the usefulness of characterizing the material properties of glycoprotein (Opell, Clouse, & Andrews, 2018). Based on this limited taxon sampling, it appears that the toughness of both flagelliform fibers and glycoprotein are phylogenetically constrained, being most similar in the two *Argiope* species, but that natural selection can more easily tune the amount of each material invested in a capture thread. Thus, the flagelliform diameters and hence work of extension of *A. trifasciata* and *A. marmoreus* are more similar than either is to *A. aurantia*. Likewise, glycoprotein volumes in *A. trifasciata* and *A. marmoreus* droplets are more similar than either is to *A. aurantia*. These quantitative similarities underlie similarities in the component contribution profiles of *A. trifasciata* and *A. marmoreus* capture threads (Figure 7).

Given differences in fly struggle behavior, it was perhaps surprising that we were able to detect and model the effect of humidity on prey retention time. However, it was probably only by eliminating spider prey capture behavior and by controlling many of the factors that affect prey retention, such as web orientation, the force with which an insect contacted capture threads, the number of threads contacted, and the part of an insect's body that initially contacted threads, that we were able to do so. Thus, our findings suggest that, even within the context of these factors, natural selection can tune the performance of viscous capture threads by directing small changes in flagelliform fiber mechanics, glycoprotein adhesion, and LMMCs composition that determines thread hygroscopicity and glycoprotein interactions.

## CONFLICT OF INTEREST

None declared.

## AUTHORS' CONTRIBUTIONS

BDO conceived the ideas, designed methodology, collected orb web samples, generated images and videos used to characterize droplet performance, performed statistical analyses, wrote the final manuscript, and prepared figures. CMB, PDD, MHYK, and MXR conducted fly retention tests, analyzed videos, performed preliminary statistical analyses, and contributed to the manuscript's first draft. HME measured droplet extension videos and constructed summary tables of values used to determine glycoprotein Young's modulus and toughness. MLH measured images and videos used to characterize droplet properties and prepared summary tables of these features. All authors gave final approval for publication, and none has a conflicting interest to declare.

## Data Availability

Primary thread property data are provided in Tables S1–S7 and fly retention time data in Table S8 (Excel).
