# Supplementary material for: Linking properties of an orb‐weaving spider's capture thread glycoprotein adhesive and flagelliform fiber components to prey retention time
Source: Ecol Evol. 2019 Aug 15;9(17):9841–54. doi: 10.1002/ece3.5525 (PMC6745672; doi:10.1002/ece3.5525)
Supplement: Supplementary file 1 [file ECE3-9-9841-s001.docx]

**Linking properties of an orb weaving spider’s capture thread glycoprotein adhesive and flagelliform fiber components to prey retention time**

Brent D. Opell, Cassandra M. Burba, Pritesh D. Deva, Matthew H. Y. Kin, Malik X. Rivas,

Hannah Mae Elmore, Mary L. Hendricks

**SUPPORTING INFORMATION – Tables S1 – S7**

**TABLE** **S1** Material properties of *A. aurantia, A. trifasciata,* and *A. marmoreus* flagelliform fibers (from Sensenig et al., 2010) and glycoproteins. Flagelliform fibers: mean ± 1 standard deviation; Glycoprotein mean ± 1 standard error.

|  | Young’s modulus  MPa | Toughness  MJ/m^3^ |
| --- | --- | --- |
| Flabelliform fibers |  |  |
| *Argiope aurantia*  Diam. 4.8 ± 1.5 µm  Work 3,818 nJ | 9 ± 11 | 211 ± 99 |
| *Argiope trifasciata*  Diam. 2.9 ± 0.9 µm  Work 1,222 nJ | 8 ± 5 | 185 ± 65 |
| *Araneus marmoreus*  Diam. 3.8 ± 1.3 µm  Work 1,849 nJ | 5 ± 2 | 163 ± 64 |
| Glycoprotein |  |  |
| *Argiope aurantia* |  |  |
| 37% RH | 0.4336 $\pm$0.3062 | 0.4209 $\pm0.1461$ |
| 55% RH | 0.0826 $\pm0.0544$ | 0.2245 $\pm0.0940$ |
| 72% RH | 0.1864 $\pm$0.1798 | 1.1013 $\pm0.9695$ |
| *Argiope trifasciata* |  |  |
| 37% RH | 0.1125 $\pm$ 0.0547 | 0.4262 $\pm$0.2545 |
| 55% RH | 0.0916 $\pm$0.0481 | 0.6252 $\pm$0.3112 |
| 72% RH | 0.0182 $\pm$ 0.0071 | 0.1252 $\pm$0.0556 |
| *Araneus marmoreus* |  |  |
| 37% RH | 0.3394 $\pm0.1751$ | 0.9288 $\pm0.5028$ |
| 55% RH | 0.2611 $\pm0.2471$ | 0.5077 $\pm0.3237$ |
| 72% RH | 0.0357 $\pm0.0216$ | 0.2088 $\pm0.0950$ |

**TABLE** **S2** Characteristics of *Argiope aurantia* droplets at three test humidities. Mean ± 1 standard error.

| Droplet Feature | Relative Humidity | | |
| --- | --- | --- | --- |
|  | 37% | 55% | 72% |
| Glyco. area & vol. No. | 14 | 14 | 11 |
| Droplet length μm | 63 ± 3.4 | 64 ± 4.1 | 67 ± 4.6 |
| Droplet width μm | 47 ± 2.5 | 48 ± 3.2 | 49 ± 3.6 |
| Droplet volume μm^3^ | 65,146 ± 10,065 | 71,139 ± 12,686 | 78,806 ± 17,186 |
| Droplet area μm^2^ | 11,217 ± 1,1111 | 12,964 ± 1,705 | 16,968 ± 3,290 |
| Droplet thickness μm | 5.61 ± 0.52 | 5.16 ± 0.51 | 4.72 ± 0.47 |
| Glycoprotein area μm^2^ | 1,667 ± 161 | 1,502 ± 195 | 2,213 ± 469 |
| Glycoprotein vol. μm^3^ | 9,713 ± 1,606 | 8,625 ± 1,586 | 10,693 ± 2,746 |
| Glyco. vol. / droplet vol. | 0.151 ± 0.009 | 0.119 ± 0.008 | 0.129 ± 0.009 |
| Glyco. area / glyco. vol. | 0.200 ± 0.019 | 0.222 ± 0.023 | 0.236 ± 0.025 |
| Extended droplets No. | 14 | 14 | 11 |
| Droplet length μm | 67 ± 4.4 | 68 ± 4.4 | 73 ± 4.9 |
| Droplet width μm | 50 ± 3.4 | 51 ± 3.4 | 55 ± 3.9 |
| Droplet volume μm^3^ | 82,082 ± 16,258 | 86,126 ± 16,474 | 106,789 ± 23,062 |
| I nferred glyco. vol. μm^3^ | 12,247 ± 2,309 | 10,766 ± 2,362 | 15,194 ± 4,473 |
| Droplet extension μm | 1,771 ± 240 | 2,796 ± 700 | 3,774 ± 948 |
| Droplet extension / glycol. vol. | 0.202 ± 0.041 | 0.368 ± 0.079 | 0.339 ± 0.066 |

**TABLE S3** Characteristics of *Argiope trifasciata* droplets at three test humidities. Mean ± 1 standard error.

| Droplet feature | Relative humidity | | |
| --- | --- | --- | --- |
|  | 37% | 55% | 72% |
| Glyco. area & vol. No. | 14 | 14 | 14 |
| Droplet length μm | 52 ± 2.8 | 54 ± 3.8 | 54 ± 3.7 |
| Droplet width μm | 39 ± 2.5 | 40 ± 2.9 | 41 ± 2.9 |
| Droplet volume μm^3^ | 37,236 ± 6,797 | 44,414 ± 9,184 | 44,998 ± 9,667 |
| Droplet area μm^2^ | 5833 ± 708 | 7734 ± 922 | 8196 ± 1,073 |
| Droplet thickness μm | 5.86 ± 0.42 | 5.04 ± 0.49 | 4.88 ± 0.43 |
| Glycoprotein area μm^2^ | 3,741 ± 491 | 5,827 ± 790 | 6,486 ± 878 |
| Glycoprotein vol. μm^3^ | 24,036 ± 4,646 | 34,078 ± 7,585 | 35,807 ± 7,725 |
| Glyco. vol. / droplet vol. | 0.63 ± 0.010 | 0.73 ± 0.016 | 0.78 ± 0.016 |
| Glyco. area / glyco. vol. | 0.633±0.101 | 0.733±0.016 | 0.781±0.016 |
| Extended droplet No. | 14 | 14 | 14 |
| Droplet length μm | 56 ± 3.6 | 54 ± 3.2 | 58 ± 3.4 |
| Droplet width μm | 40 ± 2.6 | 40 ± 2.6 | 43 ± 2.8 |
| Droplet volume μm^3^ | 43,740 ± 8,123 | 41,585 ± 6,896 | 52,556 ± 9,065 |
| Inferred glyco. vol. μm^3^ | 27,967 ± 5,268 | 31,512 ± 5,622 | 41,656 ± 7,178 |
| Droplet extension μm | 968 ± 170 | 2,720 ± 532 | 3,432 ± 444 |
| Droplet extension / glycol. vol. | 0.633 ± 0.010 | 0.733 ± 0.016 | 0.781 ±0.016 |

**TABLE** **S4** Characteristics of *Araneus marmoreus* droplets at three test humidities. Mean ± 1 standard error.

| Droplet feature | Relative humidity | | |
| --- | --- | --- | --- |
|  | 37% | 55% | 72% |
| Glyco area & vol. No. | 13 | 13 | 13 |
| Length μm | 68 ± 3.8 | 66 ± 3.2 | 73 ± 3.3 |
| Width μm | 53 ± 3.4 | 51 ± 3.0 | 58 ± 3.1 |
| Droplet volume μm^3^ | 92,548 ± 16,268 | 81,329 ± 12,601 | 114,757 ± 16,270 |
| Droplet thickness μm | 5.31 ± 0.41 | 4.41 ± 0.32 | 4.21 ± 0.33 |
| Glycoprotein area μm^2^ | 4,127 ± 1,003 | 6,476 ± 2,062 | 7,573 ± 2,688 |
| Glycoprotein vol. μm^3^ | 24,709 ± 7,620 | 30,942 ± 11,059 | 35,056 ± 13,450 |
| Ratio DV / GV | 0.25 ± 0.047 | 0.28 ± 0.070 | 0.23 ± 0.064 |
| Glyco. area / glyco. vol. | 0.200 ± 0.0149 | 0.258 ± 0.0250 | 0.259 ± 0.0243 |
| Extended droplets No. | 13 | 13 | 13 |
| Length μm | 69 ± 2.6 | 70 ± 3.1 | 72 ± 3.4 |
| Width μm | 54 ± 2.3 | 55 ± 2.6 | 57 ± 2.9 |
| Volume μm^3^ | 91,310 ± 10,699 | 96,961 ± 12,100 | 108,270 ± 15,872 |
| Inferred glyco. vol. μm^3^ | 22,326 ± 5,050 | 34,375 ± 11,907 | 35,373 ± 14,031 |
| Droplet extension μm | 2,081 ± 252 | 3,239 ± 391 | 4,244 ± 767 |
| Droplet extension / glycol. vol. | 0.146 ± 0.021 | 0.331 ± 0.107 | 0.399 ± 0.084 |

**TABLE** **S5**  *Argiope aurantia* phase 1 axial line deflection, computed force on extended droplet, and droplet length from 20% to full extension at three test humidities. Mean ± 1 standard error.

| *N* = 11-14 | | Glycoprotein  Volume µm^3^ | | Axial Line Angle $∡$ | Glycoprotein Filament Force $\mu$N | Droplet Length $\mu$m |
| --- | --- | --- | --- | --- | --- | --- |
| **Pre**  **Extension** | |  | |  |  |  |
| 37% RH | | | 12367 $\pm$ 2362 | 153 $\pm$3 | 7.371 $\pm$2.326 |  |
| 55% RH | | | 10747$\pm$2357 | 158$\pm$ 3 | 5.204$\pm$ 2.813 |  |
| 72% RH | | | 15144$\pm$4459 | 144$\pm$6 | 23.233 $\pm$8.379 |  |
| **20%**  **Extension** | |  | |  |  |  |
|  | 37% RH | 12367 $\pm$2362 | | 154$\pm$ 4 | 8.845 $\pm$ 3.486 | 288 $\pm$59 |
|  | 55% RH | 10747$\pm$2357 | | 160 $\pm$ 4 | 4.260 $\pm$1.997 | 212 $\pm$47 |
|  | 72% RH | 15144$\pm$4459 | | 146 $\pm$6 | 22.137$\pm$8.666 | 160$\pm$41 |
| **40% Extension** | |  | |  |  |  |
|  | 37% RH | 12367 $\pm$2362 | | 155$\pm$5 | 10.499$\pm$ 5.031 | 574$\pm$ 104 |
|  | 55% RH | 10747$\pm$2357 | | 162$\pm$ 4 | 3.663 $\pm$1.977 | 384$\pm$80 |
|  | 72% RH | 15144$\pm$4459 | | 153$\pm$ 6 | 13.664$\pm$ 5.572 | 312 $\pm$69 |
| **60% Extension** | |  | |  |  |  |
|  | 37% RH | 12367 $\pm$2362 | | 157$\pm$ 5 | 10.527 $\pm$ 5.360 | 863 $\pm$140 |
|  | 55% RH | 10747$\pm$2357 | | 163 $\pm$4 | 3.637$\pm$ 2.018 | 549 $\pm$ 118 |
|  | 72% RH | 15144$\pm$4459 | | 157 $\pm$ 5 | 8.511$\pm$ 3.297 | 456$\pm$ 85 |
| **80% Extension** | |  | |  |  |  |
|  | 37% RH | 12367 $\pm$2362 | | 158 $\pm$ 6 | 10.277 $\pm$ 5.459 | 1147$\pm$ 178 |
|  | 55% RH | 10747 $\pm$2357 | | 167 $\pm$ 4 | 3.129$\pm$ 2.061 | 774 $\pm$ 145 |
|  | 72% RH | 15144 $\pm$4459 | | 161 $\pm$ 4 | 4.612$\pm$ 1.961 | 615 $\pm$113 |
| **100%**  **Extension** | |  | |  |  |  |
|  | 37% RH | 12367 $\pm$2362 | | 162$\pm$ 5 | 7.333 $\pm$4.154 | 1497$\pm$ 198 |
|  | 55% RH | 10747 $\pm$2357 | | 171$\pm$3 | 1.437 $\pm$ 0.921 | 993 $\pm$167 |
|  | 72% RH | 15144$\pm$4459 | | 164 $\pm$4 | 3.294 $\pm$ 1.611 | 734 $\pm$ 146 |

**TABLE** **S6**  *Argiope trifasciata* phase 1 axial line deflection, computed force on extended droplet, and droplet length from 20% to full extension at three test humidities. Mean ± 1 standard error.

| *N* = 14 | | Glycoprotein  Volume µm^3^ | | Axial Line Angle $∡$ | Glycoprotein Filament Force $\mu$N | Droplet Length $\mu$m |
| --- | --- | --- | --- | --- | --- | --- |
| **Pre**  **Extension** | |  | |  |  |  |
| 37% RH | | | 27967 $\pm$ 5268 | 136 $\pm$ 5 | 12.033$\pm$ 4.669 |  |
| 55% RH | | | 31512 $\pm$ 5622 | 129 $\pm$4 | 13.271$\pm$ 2.919 |  |
| 72% RH | | | 41656 $\pm$7178 | 133$\pm$ 4 | 9.774 $\pm$ 2.087 |  |
| **20%**  **Extension** | |  | |  |  |  |
|  | 37% RH | 27967 $\pm$ 5268 | | 139$\pm$ 5 | 9.980 $\pm$3.845 | 244 $\pm$ 41 |
|  | 55% RH | 31512$\pm$ 5622 | | 146$\pm$ 4 | 5.859$\pm$ 1.982 | 617 $\pm$ 69 |
|  | 72% RH | 41656 $\pm$7178 | | 150$\pm$ 4 | 3.794$\pm$1.407 | 542 $\pm$ 93 |
| **40% Extension** | |  | |  |  |  |
|  | 37% RH | 27967 $\pm$ 5268 | | 141 $\pm$4 | 6.938 $\pm$ 1.503 | 438 $\pm$ 85 |
|  | 55% RH | 31512 $\pm$ 5622 | | 153$\pm$5 | 4.499 $\pm$ 1.861 | 878 $\pm$ 90 |
|  | 72% RH | 41656$\pm$7178 | | 157 $\pm$ 3 | 2.445$\pm$ 1.048 | 735 $\pm$ 92 |
| **60% Extension** | |  | |  |  |  |
|  | 37% RH | 27967 $\pm$ 5268 | | 142 $\pm$4 | 6.539 $\pm$1.193 | 588 $\pm$ 117 |
|  | 55% RH | 31512 $\pm$ 5622 | | 158 $\pm$5 | 3.237$\pm$ 1.534 | 1120 $\pm$ 99 |
|  | 72% RH | 41656$\pm$7178 | | 162 $\pm$3 | 2.067$\pm$ 1.070 | 877 $\pm$ 87 |
| **80% Extension** | |  | |  |  |  |
|  | 37% RH | 27967$\pm$ 5268 | | 144 $\pm$ 4 | 6.032 $\pm$1.054 | 737 $\pm$ 148 |
|  | 55% RH | 31512$\pm$ 5622 | | 162 $\pm$5 | 2.476$\pm$1.352 | 1316 $\pm$ 109 |
|  | 72% RH | 41656 $\pm$7178 | | 165 $\pm$ 3 | 1.847 $\pm$ 1.091 | 996 $\pm$ 85 |
| **100%**  **Extension** | |  | |  |  |  |
|  | 37% RH | 27967 $\pm$ 5268 | | 146 $\pm$4 | 5.218$\pm$0.958 | 935 $\pm$170 |
|  | 55% RH | 31512 $\pm$ 5622 | | 166$\pm$ 4 | 1.679 $\pm$ 1.087 | 1531 $\pm$121 |
|  | 72% RH | 41656 $\pm$7178 | | 168 $\pm$ 3 | 1.498$\pm$0.916 | 1090 $\pm$91 |

**TABLE** **S7**  *Araneus marmoreus* phase 1 axial line deflection, computed force on extended droplet, and droplet length from 20% to full extension at three test humidities. Mean ± 1 standard error.

| *N* = 11- 14 | | Glycoprotein  Volume µm^3^ | | Axial Line Angle $∡$ | Glycoprotein Filament Force $\mu$N | Droplet Length $\mu$m |
| --- | --- | --- | --- | --- | --- | --- |
| **Pre**  **Extension** | |  | |  |  |  |
| 37% RH | | | 24683 $\pm$ 6758 | 145 $\pm$4 | 6.260 $\pm$2.430 |  |
| 55% RH | | | 39390$\pm$14318 | 146 $\pm$4 | 4.820 $\pm$ 1.629 |  |
| 72% RH | | | 30806$\pm$10739 | 151 $\pm$3 | 3.125$\pm$1.081 |  |
| **20%**  **Extension** | |  | |  |  |  |
|  | 37% RH | 24683 $\pm$6758 | | 148$\pm$ 5 | 5.295 $\pm$ 2.441 | 441 $\pm$ 38 |
|  | 55% RH | 39390 $\pm$14318 | | 157$\pm$ 4 | 1.768 $\pm$ 0.607 | 492 $\pm$ 54 |
|  | 72% RH | 30806$\pm$10739 | | 158$\pm$3 | 1.825$\pm$ 0.601 | 299 $\pm$ 41 |
| **40% Extension** | |  | |  |  |  |
|  | 37% RH | 24683 $\pm$ 6758 | | 148 $\pm$ 5 | 6.070 $\pm$ 2.713 | 745$\pm$ 61 |
|  | 55% RH | 39390$\pm$14318 | | 160 $\pm$4 | 1.435$\pm$0.594 | 781 $\pm$ 67 |
|  | 72% RH | 30806$\pm$10739 | | 161$\pm$3 | 1.538 $\pm$ 0.499 | 482 $\pm$ 61 |
| **60% Extension** | |  | |  |  |  |
|  | 37% RH | 24683 $\pm$ 6758 | | 148 $\pm$5 | 6.195$\pm$ 2.699 | 1090 $\pm$ 89 |
|  | 55% RH | 39390$\pm$14318 | | 164$\pm$4 | 1.263$\pm$0.716 | 1068 $\pm$ 80 |
|  | 72% RH | 30806$\pm$10739 | | 163$\pm$3 | 1.180$\pm$ 0.392 | 666 $\pm$ 102 |
| **80% Extension** | |  | |  |  |  |
|  | 37% RH | 24683 $\pm$ 6758 | | 149$\pm$6 | 6.095$\pm$ 2.750 | 1459 $\pm$ 115 |
|  | 55% RH | 39390$\pm$14318 | | 166 $\pm$4 | 1.161 $\pm$ 0.716 | 1319 $\pm$ 112 |
|  | 72% RH | 30806 $\pm$10739 | | 166$\pm$ 3 | 0.993 $\pm$ 0.457 | 866 $\pm$135 |
| **100%**  **Extension** | |  | |  |  |  |
|  | 37% RH | 24683$\pm$ 6758 | | 154 $\pm$6 | 4.766 $\pm$2.467 | 1940$\pm$150 |
|  | 55% RH | 39390 $\pm$14318 | | 169 $\pm$4 | 0.664 $\pm$ 0.427 | 1593$\pm$ 169 |
|  | 72% RH | 30806 $\pm$10739 | | 167 $\pm$3 | 0.926$\pm$ 0.439 | 1023$\pm$ 162 |
